# Acute COVID-19 Syndrome Predicts Severe Long COVID-19: An Observational Study

**DOI:** 10.7759/cureus.29826

**Published:** 2022-10-02

**Authors:** Antonio S Menezes, Silvia M Botelho, Luciana R Santos, Aline L Rezende

**Affiliations:** 1 Cardiology, Pontifical Catholic University of Goiás, Goiânia, BRA; 2 Internal Medicine/Cardiology, Federal University of Goiás, Goiânia, BRA; 3 Internal Medicine, Federal University of Goiás, Goiânia, BRA; 4 Internal Medicine, Pontifical Catholic University of Goiás, Goiânia, BRA

**Keywords:** sars-cov-2., predictors, observational study, long covid-19, laboratory findings

## Abstract

Introduction

Tissue damage, chronic dysfunction, and symptoms that last more than 12 weeks are hallmarks of long-term chronic opportunistic viral infection (COVID-19), and the disease may have a permanent, relapsing/remitting, or gradually improving course. This study aimed to determine the risk factors of severe long COVID-19.

Methods

In October 2021, primary care clinics enrolled consenting 18- to 89-year-olds to complete an online questionnaire on self-diagnosis, clinician diagnosis, testing, symptom presence, and duration of COVID-19. Long COVID-19 was identified if symptoms were beyond 12 weeks. Patients with long-lasting COVID-19 symptoms were assessed using multivariable regression to identify potential predictors of severe long COVID-19.

Results

Of the 220 respondents, 108 (49%) patients were self- or clinician-diagnosed with COVID-19 or had a confirmed positive laboratory test result. Patients aged >45 years and with at least 15 COVID-19 symptoms were 5.55 and 6.02 times, respectively, more likely to acquire severe long COVID-19. Most patients with severe and moderate post-acute COVID-19 syndrome had no relevant comorbidities (p=0.0402; odds ratio [OR]=0.4; 95% confidence interval [CI]=0.18-0.98). Obesity was a significant predictor (p=0.0307; OR=6.2; 95% CI=1.1-33.2).

Conclusion

The simultaneous presence of 15 or more COVID-19 symptoms, age >45 years, and obesity were related to a higher probability of severe long COVID-19.

## Introduction

COVID-19 is caused by a severe acute respiratory syndrome coronavirus 2 (SARS-CoV-2) infection, which may be asymptomatic or may induce a broad spectrum of symptoms, including features of mild upper respiratory tract infection and life-threatening sepsis. Until October 25, 2021, 244 million SARS-CoV-2 infections and 5 million COVID-19-related deaths were reported [1]. Symptoms of acute SARS-CoV-2 infection include fever, lethargy, myalgia, fatigue, headache, rhinorrhea, dry cough, dyspnea, smell or taste alterations, nausea, vomiting, and diarrhea [1,2]. On exposure to SARS-CoV-2, the body mounts a rapid immunological response, which is characterized by increased levels of chemokines, proinflammatory cytokines, and activated monocytes. Subsequently, immunoglobulin M (IgM), IgA, and IgG antibodies and interferon-producing T cells are produced [2-7], and this immune response suppresses replication of SARS-CoV-2, which becomes non-infectious after three weeks, and this corresponds with recovery from COVID-19 [8,9].

Approximately one-third of COVID-19 patients experience the post-acute COVID-19 syndrome (PACS), wherein symptoms persist for 4-12 weeks after the initial SARS-CoV-2 infection [2,9]. PACS differs from the persistent post-COVID-19 syndrome, wherein symptoms (called sequelae) last for >84 days after COVID-19 [10]. Depending on the definition and level of patient care, the prevalence of PACS was 10%-60% in all COVID-19 hospitalized cases [11,12]. PACS symptoms include fatigue, dyspnea, and cognitive impairment (“brain fog”). Therefore, early detection of SARS-CoV-2 infection is important in at-risk individuals because changes the management or outcome of PACS.

Furthermore, 10%-15% of all COVID-19 patients may present persistent symptomatology weeks or even months after the initial infection. Given the cumulative burden of COVID-19 in Catalonia, Spain, we speculate that over 90,000 patients could have been or are currently affected by persistent symptoms or sequelae. Several definitions of PACS have been suggested [11-14].

The post-COVID-19 syndrome can be classified into two subtypes: post-acute COVID-19 with persistent symptoms lasting beyond four weeks after infection, with a permanent, relapsing/remitting or progressively improving course (PACS) and long COVID-19 with irreversible tissue damage that lasts beyond 12 weeks, which causes persistent malfunction and symptoms (sequelae) [10-14]. In younger patients, among whom the incidence of PACS is 8%, the multisystemic inflammatory syndrome is the result of an abnormal immune response, which although uncommon, may be dangerous and requires early diagnosis and treatment. Fatigue (52%), cardiorespiratory (30%-42%) and neurological (40%) symptoms are the most common features in younger patients [9,11-13].

Nonetheless, the patient’s age and number of symptoms during the first week of illness may predict the risk for PACS [14,15]. Self-reported data and telehealth surveys can be biased, and the presence of SARS-CoV-2 risk factors impedes the identification of PACS risk variables independently of disease severity. Therefore, this study aimed to determine the clinical factors that may accurately predict severe long COVID-19.

## Materials and methods

Study population and research design

This prospective observational study was conducted in Goiânia, Brazil, from February 2021 to March 2022, through both clinical evaluation and completion of a self-administered questionnaire among patients who were previously infected with SARS-CoV-2 and presented with acute symptoms.

This study was approved by the Research Ethics Committee, according to the National Health Council Resolution 466/2012, and was performed in accordance with the principles underlying the Declaration of Helsinki (CAAE number 47544021.9.0000.0037). The eligibility criteria were age >18 years and previously tested positive for SARS-CoV-2 (positive oropharyngeal or pharyngeal nasal swab test by reverse transcription-polymerase chain reaction [RT-PCR]).

One hundred eight individuals who had SARS-CoV-2 infections during the pandemic and realized any diagnosis as a prior protocol of COVID-19 were included in the study population and participated in the research (RT-PCR, serological examination, and chest CT scans). Every participant filled out a questionnaire and gave written informed permission to participate in the research. They also received a clinical assessment and answered questions about the clinical signs of the illness. The period for data collection was from June through October 2021.

During the clinical evaluation, the demographics, including age, sex, and time since the initial infection, were recorded. Information about the acute phase of infection, such as disease severity, pulmonary involvement, clinical management, and pre-existing comorbidities, were collected. The presence or absence of PACS alterations was determined via the questionnaire.

In the current study, patients who exhibited symptoms but did not have evidence of major viral pneumonia or hypoxemia were classified as having moderate COVID-19. On the other hand, patients who exhibited clinical signs of pneumonia in conjunction with iron-deficiency anemia or hypoxemia, acute respiratory distress syndrome, sepsis, thromboembolic events, or systemic inflammatory response syndrome were classified as having severe COVID-19. Chest computed tomography was used to determine the presence of pulmonary involvement (CT).

Statistical analysis

The power was estimated for the sample n established power result (1- err prob) = 0.87 or 87%, considering the significance limit of 5% (= 0.05) with a mean effect size of 0.6, considering the type of statistical analysis. With this, the sample size was sufficient to determine the results obtained. The sample calculation was performed with the GPower software version 3.1.

Most of the characteristics associated with the COVID-19 response consisted of dichotomous information; therefore, binary logistic regression analysis was applied. Thus, in this study, the 23 predictor variables (symptoms and comorbidities) were coded as 1 or 0, which corresponded to the presence or absence, respectively, of the characteristic in the patient.

In the binary logistic regression model, constructed using SPSS Statistics for Windows (version 27.0; IBM Corp., Armonk, NY), the stepwise variable-filtering criterion was applied. Briefly, the model started with 23 variables; a variable was eliminated in each step until the final model was constructed with the minimum number of variables that had the best statistical significance to determine the probability of severe long COVID-19. The statistical significance criterion adopted for the entry of variables was p≤0.10, and that for the output was p≤0.05.

These qualitative variables are presented as absolute and relative frequencies and as the measures of central tendency with a variation. In the inferential analysis, the following methods were applied: (a) in the bivariate analyses, the OR test followed by the 2-tailed test was applied, and Fisher’s exact test was performed; and (b) variables with p<0.05, when related to severe long COVID-19, were included in the multivariate logistic regression model with a regressive stepwise criterion. The alpha error was previously fixed at 5% for the rejection of the null hypothesis, and statistical processing was performed using BioEstat version 5.3 and SPSS version 27 (IBM).

## Results

We analyzed data from 108 people aged 18-89 years, with a mean age of 43 ± 15 years. There were 68 (63%) women and 40 (37%) men in this study. These patients were cared for either at home (n = 73, 67.6%), in the hospital (n = 9, 8.3%), or in the intensive care unit (n = 26, 24.1%). COVID-19 was diagnosed using RT-PCR in 97.25% of cases; the remaining cases were diagnosed using IgG+ (2) and CT imaging (1).

Patients with moderate and severe acute COVID-19

To develop a prediction model of severe long COVID-19, the symptoms, and comorbidities of 108 patients were analyzed, and 23 characteristics with p<0.05 in the bivariate odds ratio (OR) analysis were included in the binary logistic regression model. These variables are marked with asterisks in Tables 1-3. Comparisons of sex, age, time of infection, and impairment detected on CT during the period of disease manifestation demonstrated that age ≥45 years (OR = 4.9) and >50% pulmonary involvement were significantly correlated with severe long COVID-19 (Table 1).

**Table 1:** Classification of COVID-19 patients *Chi-square test was performed to compare the odds ratios. CI: confidence interval; NNH: number needed to harm; OR: odds ratio.

	Severe COVID-19		Moderate COVID-19					
	n	%	n	%	OR	95% CI	NNH	p-value
Sex, male	17	50.0	23	31.1	2.2	0.9–5.1	6	0.0937
Age (≥45 years) *	23	67.6	22	29.7	4.9	9- 11.8	3	0.0005*
Time of infection (≥4 days)	18	52.9	30	40.5	1.5	0.7–3.7	9	0.3192
Pulmonary involvement (≥50%)	34	100.0	0	0.0	2625	---	2	<0.0001*

Symptoms in acute COVID-19

The post-COVID-19 changes evaluated in this study were related to cardiovascular disorders (arrhythmia, myocarditis, new systemic arterial hypertension, increased resting heart rate, and palpitations), respiratory disorders (cough, chest pain, sleep apnea, post-activity apnea, dyspnea, and sputum), neurological symptoms (headache, memory loss, and attention disorders), nausea and vomiting, sleep disorders, dizziness, ageusia, psychiatric disorders (anxiety, depression, obsessive-compulsive disorder, mood disorders, dysphoria, paranoia, mental health changes, and psychiatric illnesses), integumentary symptoms (hair loss and skin signs), and general or non-specific symptoms (fatigue, arthralgia, general pain, fever, sweating, gastrointestinal tract disorders, weight loss, chills, hearing loss or tinnitus, red eyes, edema, sore throat, renal failure, and diabetes mellitus), as seen in Table 2.

**Table 2:** Distribution of symptoms in patients with severe and moderate COVID-19 *Chi-square test performed for the odds ratio. **Fisher’s exact test. CI: confidence interval HR: heart rate; NNH: number needed to harm; OCD: obsessive-compulsive disorder; OR: odds ratio; SAH: systemic arterial hypertension. Of the abovementioned 23 factors, the factors that demonstrated a significant association with severe long COVID-19 are denoted by a superscript hash sign (#).

	Severe COVID-19		Moderate COVID-19					
Symptoms	n	%	n	%	OR	95% CI	NNH	p-value
Fatigue^#^	27	79.4	39	52.7	3.4	1.3–8.9	4	0.0150*
Headache	18	52.9	25	33.8	2.2	0.9–5.1	6	0.0935
Attention disorder	18	52.9	32	43.2	1.4	0.6–3.3	11	0.4648
Hair loss^#^	23	67.6	29	39.2	3.2	1.3–7.6	4	0.0011*
Dyspnea^#^	20	58.8	20	27.0	3.8	1.6–9.1	4	0.0030*
Ageusia^#^	14	41.2	12	16.2	3.6	1.4–9.1	5	0.0100*
Anosmia	12	35.3	15	20.3	0.9	0.35–2.3	46	0.9464
Polypnea	28	82.4	49	66.2	2.3	0.8–6.5	7	0.1355
Arthralgia	14	41.2	22	29.7	1.6	0.7–3.8	9	0.3413
Cough^#^	18	52.9	9	12.2	8.1	3.1–21.4	3	<0,0001*
Sweat^#^	16	47.1	13	17.6	4.1	1.7–10.2	4	0.0029*
Nausea/vomiting	11	32.4	12	16.2	2.4	0.9–6.3	7	0.0991
Memory loss^#^	21	61.8	30	40.5	2.4	1.03–5.4	5	0.0402*
OCD^#^	8	23.5	2	2.7	11.1	2.2–55.8	5	0.0019**
Anxiety	25	73.5	53	71.6	1.1	0.4–2.7	53	0.9795
Gastrointestinal disorders	12	35.3	23	31.1	1.2	0.5–2.8	24	0.8312
Weight loss^#^	13	38.2	14	18.9	2.6	1.1–6.5	6	0.0313*
Cutaneous signs^#^	10	29.4	8	10.8	3.4	1.2–9.7	6	0.0331*
Palpitations	21	61.8	40	54.1	1.4	0.6–3.1	13	0.5883
General pain	11	32.4	20	27.0	1.29	0.5–3.1	19	0.7344
Fever^#^	6	17.6	0	0.0	16.1	1.8–139	7	0.0007**
Sleep disorders	20	58.8	34	45.9	1.6	0.7–3.8	8	0.3002
Sleep apnea^#^	10	29.4	3	4.1	9.8	2.5–38.8	4	0.0006*
Chills	11	32.4	14	18.9	2.1	0.8–5.1	8	0.1965
Psychiatric disorders^#^	9	26.5	4	5.4	6.3	1.7–22.8	5	0.0050*
Red eyes	9	26.5	18	24.3	1.1	0.4–2.8	47	0.9999
Auditory loss or tinnitus	13	38.2	15	20.3	2.4	0.9–5.9	6	0.0815
Mental illness	7	20.6	8	10.8	2.1	0.7–6.4	11	0.2869
Diabetes^#^	7	20.6	0	0.0	19.2	2.2–163	6	0.0002*
Sputum^#^	11	32.4	11	14.9	2.7	1.04–7.1	6	0.0361*
Edema	9	26.5	11	14.9	2.1	0.7–5.5	9	0.2398
Dizziness	19	55.9	19	25.7	3.6	1.5–8.6	4	0.0046*
Sore throat	6	17.6	12	16.2	1.1	0.3–3.2	70	0.9262
Mood disorder^#^	19	55.9	22	29.7	2.9	1.3–6.9	4	0.0170*
Dysphoria^#^	8	23.5	3	4.1	7.8	1.8–29.5	6	0.0039**
Myocarditis^#^	5	14.7	0	0.0	12.7	1.4–113	8	0.0110**
Renal failure^#^	9	26.5	0	0.0	26.6	3.2–220	4	0.0001*
Arrhythmia^#^	17	50.0	7	9.5	9.5	3.4–26.7	3	<0.0001*
Paranoia^#^	8	23.5	0	0.0	22.7	2.7–190	5	0.0001**
Depression^#^	15	44.1	15	20.3	3.1	1.2–7.5	5	0.0194*
Chest pain	21	61.8	40	54.1	1.4	0.5–3.1	13	0.5882
New SAH*	11	32.4	9	12.2	3.4	1.2–9.3	5	0.0250*
Increased HR at rest	19	55.9	37	50.0	1.2	0.6–2.8	17	0.7182
≥15 symptoms *	25	73.5	17	23.0	9.3	3.6–23.7	2	<0.0001*

Pre-existing comorbidities of COVID-19 patients

A significant number of patients with severe and moderate COVID-19 had no notable comorbidity (p=0.0402; OR 0.4; 95% CI 0.18-0.98). Moreover, significantly more obese patients had severe or moderate COVID-19 (p=0.0307; OR 6.2; 95% CI 1.1-33.2; Table 3).

**Table 3:** Pre-existing comorbidities in patients with acute COVID-19 *Chi-square test applied on a 2×2 odds ratio table. **Fisher’s exact test CI: confidence interval; COPD: chronic obstructive pulmonary disease; NNH: number needed to harm; OR: odds ratio; SAH: systemic arterial hypertension. Statistically significant data are denoted by a superscript hash sign (#).

	Severe COVID-19		Moderate COVID-19									
Comorbidities	N	%	n	%	OR	95% CI		NNH		p-value		
None^#^	13	38.2	44	59.5	0.4	0.18–0.98		---		0.0402*		
Bronchial asthma	0	0.0	1	1.4	2.7	---		67		0.9999		
Chagas disease	1	2.9	6	8.1	0.3	0.03–2.9		---		0.429		
Coronary arteriopathy	2	5.9	3	4.1	1.5	0.2–9.2		55		0.9975		
Dyslipidemia	3	8.8	3	4.1	2.3	0.4–11.9		21		0.3764		
Diabetes	2	5.9	3	4.1	1.5	0.2–9.2		55		0.9975		
COPD	2	5.9	0	0.0	4.3	0.3–49.0		24		0.1051		
SAH	8	23.5	15	20.3	1.2	0.4–3.2		31		0.8956		
Obesity^#^	5	14.7	2	2.7	6.2	1.1–33.2		9		0.0307**		

The statistical results from the stepwise regression method were applied to the 23 variables related to severe long COVID-19. Only two variables demonstrated significance (p<0.05): age ≥45 years and the presence of ≥15 symptoms. Therefore, we found sufficient evidence that these variables influence the exacerbation of COVID-19, as seen in Table 4.

**Table 4:** Variables affecting COVID-19 aggravation *Logistic regression. X/σ: Z-score which indicates the likelihood that a given observation is derived from normally distributed data (with mean zero). B: binomial; CI: confidence interval; OR: odds ratio; SE: sensitivity; Z: Wald test.

	B	SE	Wald	Z	p-value	OR	95% CI	
Age ≥45 years	1.715	0.628	7.446	2.72	0.006*	5.55	1.6–19.0	
Fever	10.937	58.7	0.000	0.18	0.852	1.00	0.000–+∞	
Renal failure	11.787	42.7	0.000	0.27	0.782	1.00	0.000–+∞	
Paranoia	11.952	45.7	0.000	0.26	0.998	1.00	0.000–+∞	
≥15 symptoms	1.795	0.615	8.512	2.91	0.004*	6.02	1.8–20.1	
Constant	−3.103	0.607	26.109		0.0001*	0.04		

**Figure 1:**
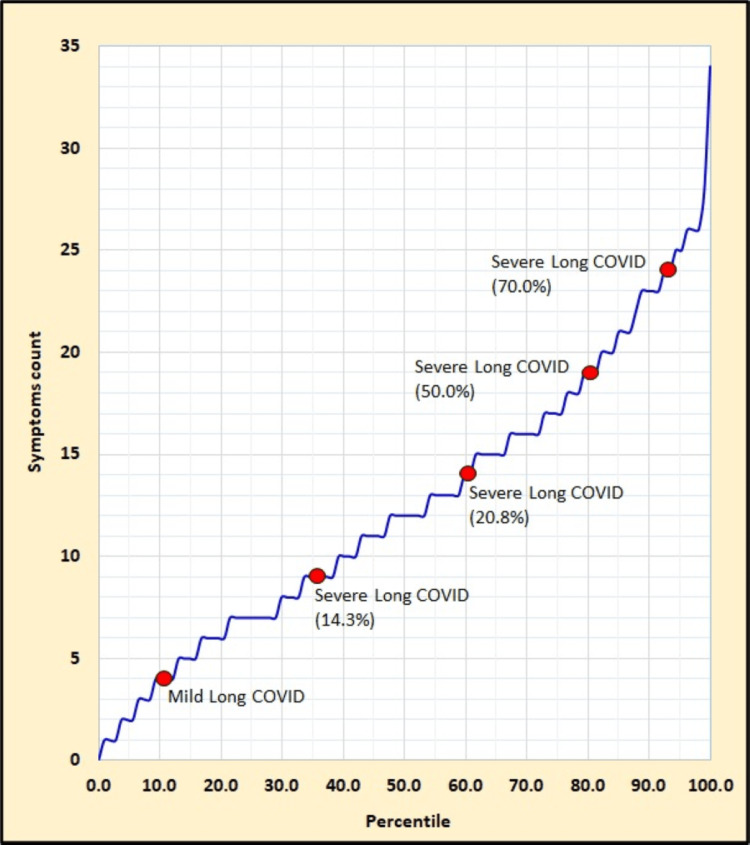
Correlation between symptoms and long COVID-19 severity

According to the prediction model, patients aged ≥45 years have a 5.55-fold greater likelihood of developing severe long COVID-19 than younger patients. The coefficient estimates are based on a comparison made between the two age groups. Furthermore, patients who exhibited ≥15 symptoms had a 6.02-fold increased risk of developing severe long COVID-19 than those who exhibited <15 symptoms (Figure 1).

## Discussion

The SARS-CoV-2 virus has a variety of impacts on people across the world, depending on factors such as heredity, the environment, location, and the dynamics of the population [12]. This study profiled a large cohort that showed signs of long-term effects of COVID-19.

Although there is no significant difference in susceptibility to SARS-CoV-2 between sexes, the number of post-COVID-19 symptoms in females is slightly higher than that in males although males have greater severity and higher mortality rates with COVID-19 [10,13]. The inflammatory response is dysregulated and is likely more activated in males [13], which may explain the increased severity of persistent symptoms following COVID-19.

Age is closely related to the severity of post-COVID-19 symptoms [13], and age >45 years is a predictor of severe long COVID-19 after the acute phase [3]. The present study shows that patients ≥45 years had significantly more post-COVID-19 symptoms than younger patients, which may be due to the correlation between greater symptom severity during the acute phase and the higher number of chronic symptoms.

The disease severity and extent of its spread in the lungs are proportionate to the number of chronic symptoms of the patient. Patients who were admitted to the intensive care unit and required mechanical ventilation had significantly more symptoms than those with a mild form of the disease, who were admitted to the general ward, or who were treated at home. This is analogous to patients who have had moderate or severe COVID-19 in which the symptoms persisted for significantly longer than those who had a mild form of the disease. This is thought to be partially caused by structural and functional damage to the main organ systems (cardiopulmonary, neurological, and renal), including pulmonary fibrosis, thromboembolic events, inflammation of the central nervous system, myocardial inflammation, arrhythmias, and acute kidney injury [6,9,14-20].

Studies on post-COVID-19 manifestations have shown associations between severe illness during the acute phase and severe long-term symptoms [14]. In patients with severe long COVID-19, chronic inflammation has been observed in the lungs, heart, kidneys, liver, pancreas, and spleen based on imaging investigations [2,5,6]. Patients with severe COVID-19 have elevated levels of proinflammatory cytokines during the acute phase of infection. These cytokines continue to be present at elevated levels in the blood throughout the chronic phase of infection, which further results in chronic symptoms [6]. For COVID-19, the acute inflammatory response is responsible for the tissue damage that plays a crucial role in disease progression [7,15-17,21].

In addition, “post-intensive treatment syndrome” plays a significant role in the development of long-term sequelae after COVID-19 [21]. Prolonged periods of immobility, administration of massive quantities of sedatives and neuromuscular blockers, respiratory shock, and viral toxicity could lead to numerous outcomes of physical restriction, such as fatigue and chronic myalgia. Furthermore, these factors could lead to psychological symptoms, such as anxiety, depression, and post-traumatic stress disorder, along with neurological symptoms, such as memory loss, attention deficiency, and delirium, which may be permanent in the most severe cases [19-21].

Moderate manifestations of COVID-19 during the acute phase do not necessarily result in a reduced likelihood of developing chronic PACS [22]. Similarly, although comorbidities influence disease progression during the acute phase, they do not appear to influence post-COVID-19 symptoms [22]. There was a significant difference in the number of post-COVID-19 symptoms observed between patients with comorbidities and those who were obese but did not have any other comorbidities.

There was no significant correlation between the time elapsed since infection and the intensity of post-COVID-19 symptoms, which indicated that symptoms can continue to emerge for more than a year after the initial infection. There is currently no documented time scale that corresponds to the commencement of the disappearance of symptoms [21]. However, as COVID-19 is a relatively new phenomenon, it is too early to determine how long these chronic symptoms will continue to exist and whether they are permanent or temporary sequelae.

The cardiovascular, pulmonary, neurological, and muscular systems have been implicated in most post-COVID-19 symptoms, as documented through various trials [2-6,22]. Long-term investigations into individuals who had survived severe respiratory SARS-CoV-2 infection show psychological morbidities and clinically significant persistent fatigue for up to four years following the acute respiratory phase of the disease [7,9]. This phenomenon may be caused by high serum levels of cortisol and proinflammatory cytokines that persist after the acute phase, whereas psychiatric disorders are believed to be caused by the trauma resulting from the severity of the respiratory condition and the socioeconomic and cultural impacts of the long recovery process [5,7].

In addition, some of these individuals have shown symptoms of post-febrile chronic fatigue syndrome, widespread myalgia, muscular weakness, and sleep disorders [23]. As the progression of SARS-CoV-2 infection follows the same pattern as that of SARS infection [24], it is speculated that high incidences of long-term psychiatric disorders and chronic fatigue are caused by the same mechanisms that were previously observed in SARS and that these symptoms may last for long periods. There are reports of patients with PACS suffering from chronic exhaustion for more than 100 days, and this is the primary symptom of the clinical condition’s deterioration [15-20,25-27].

By colonizing the upper airways, the virus also colonizes the olfactory bulb and accesses the central nervous system to cause persistent neuronal inflammation, which may be responsible for SARS-CoV and SARS-CoV-2-associated neurological disorders. In addition, autopsy studies of SARS-CoV-2 colonization of the central nervous system indicate that the virus possibly colonizes the cerebrovascular endothelium and cerebral parenchyma to reach neurons and glial cells, particularly in the medial temporal lobe, which results in apoptosis and necrosis. In addition, tissue damage caused by the systemic inflammatory response [15-17,27] contributes to persistent damage to the central nervous system. Therefore, the neurological symptoms associated with SARS and COVID-19 may persist for indeterminate periods of time or may be permanent. This may partly explain why there is no significant relationship between the time since infection and the severity of post-COVID-19 symptoms.

More than half of the individuals who participated in another COVID-19 study presented with sleep disturbances [25,26], which may be a consequence of the direct neurological damage that is caused by the virus when it crosses the blood-brain barrier. Sleep disturbances can lead to circadian cycle abnormalities because of the systemic inflammatory response and psychological illnesses induced by the infection [18]. After the onset of COVID-19, patients may experience cardiovascular changes such as palpitations, an elevated resting heart rate, chest discomfort, myocarditis, and arrhythmias [6,19], which is mostly because of prolonged myocardial inflammation. Palpitations, ventricular arrhythmias, and sinus or supraventricular tachyarrhythmias were the primary cardiovascular abnormalities observed in this study and are comparable with those described earlier [22]. Pulmonary involvement, which is indicated by high rates of post-activity polypnea (71.6%) and dyspnea (37.6%), is primarily attributable to the lower diffusion capacity that results from viral involvement and pulmonary scarring-related fibrosis [20]. Furthermore, cardiovascular sequelae may play an important role in the genesis of respiratory sequelae, as cardiovascular sequelae may lead to pulmonary hypertension owing to a decline in left ventricular function [20]. Similar to other studies [26-28], a connection between cardiovascular and pulmonary symptoms was identified in this study.

Hair loss was the most prevalent symptom associated with the integumentary system (48.2%) and was most likely caused by PACS [18]. In most cases of post-COVID-19 telogen effluvium (TE), hair loss was documented [19]. This is because elevated IL-6 suppresses the proliferation of matrix cells inside hair follicles. Besides inducing excessive fluoride production in the telogen phase, COVID-19 may aggravate pre-existing abnormalities of fluoride production. Moreover, the stress induced by the pandemic as well as the illness itself is a risk factor for TE [20].

In the present study, the duration of symptoms associated with PACS could not be determined as the patients in the trial were not followed for adequate time to document the disappearance of symptoms. In addition, some patients reported having symptoms, but there was no clinical evidence to support the existence of those symptoms.

Limitations

Non-response bias can affect questionnaire-based studies, and the patients surveyed may not be representative of the overall population. The electronic format may have hampered the participation of older and physically frail respondents. Responses from black, Asian, and other ethnic groups may have been underrepresented. Long-term symptom sufferers may over-report their symptoms whereas those with more symptoms may have reported only persistent symptoms, which may have accentuated the differences in symptom features between long COVID-19 and long-term or acute COVID-19. Finally, the length of COVID-19-related symptoms depends on the patient’s recall. Thus, it could not be determined whether the symptoms were acute or if they persisted in the long term.

## Conclusions

The diagnostic parameters of COVID-19 indicate that there may be several independent underlying correlations between the symptoms, which may influence symptom severity or development. The present study revealed that patients who did not exhibit any comorbidities and were obese were at significant risk of developing severe long COVID-19. The number of symptoms present (>15 symptoms), age >45 years, and obesity in COVID-19 patients were predictors of severe long COVID-19. The identification of these and other factors that contribute to severe long COVID-19 is key to supporting these patients.
